# Jamming Strategy Optimization through Dual Q-Learning Model against Adaptive Radar

**DOI:** 10.3390/s22010145

**Published:** 2021-12-26

**Authors:** Hongdi Liu, Hongtao Zhang, Yuan He, Yong Sun

**Affiliations:** Key Lab of Universal Wireless Communications, Ministry of Education of China, Beijing University of Posts and Telecommunications, Beijing 100876, China; liuhongdi@bupt.edu.cn (H.L.); yuanhe@bupt.edu.cn (Y.H.); sunyong@bupt.edu.cn (Y.S.)

**Keywords:** adaptive radar, jamming strategy optimizing, reinforcement learning, Q-learning, jamming effectiveness evaluation

## Abstract

Modern adaptive radars can switch work modes to perform various missions and simultaneously use pulse parameter agility in each mode to improve survivability, which leads to a multiplicative increase in the decision-making complexity and declining performance of the existing jamming methods. In this paper, a two-level jamming decision-making framework is developed, based on which a dual Q-learning (DQL) model is proposed to optimize the jamming strategy and a dynamic method for jamming effectiveness evaluation is designed to update the model. Specifically, the jamming procedure is modeled as a finite Markov decision process. On this basis, the high-dimensional jamming action space is disassembled into two low-dimensional subspaces containing jamming mode and pulse parameters respectively, then two specialized Q-learning models with interaction are built to obtain the optimal solution. Moreover, the jamming effectiveness is evaluated through indicator vector distance measuring to acquire the feedback for the DQL model, where indicators are dynamically weighted to adapt to the environment. The experiments demonstrate the advantage of the proposed method in learning radar joint strategy of mode switching and parameter agility, shown as improving the average jamming-to-signal radio (JSR) by 4.05% while reducing the convergence time by 34.94% compared with the normal Q-learning method.

## 1. Introduction

In radar jamming, accurate decision-making is an important prerequisite for effective jamming. For single-mode radar with only a few fixed parameter combinations, the jamming decision-making based on template matching can be efficient [1]. Nowadays, with the improvement of electronic technology, modern radars tend to be adaptive. Adaptive radars can perform various tasks through automatic work mode switching, where work modes vary with different radars. Take a ground-based radar with search, tracking, and recognition modes as an example: The radar initially scans the entire airspace at the search mode, and switches to the tracking mode when a mission-related target is detected, then transitions to the recognition mode after the target is confirmed. If the echo signal quality drops or the target is lost due to the jamming, the radar will take anti-jamming measures autonomously or return to the search mode. For phased array radars with modes named “range while scan” (RWS), “track and search” (TAS), “Single Target Track” (STT), etc., the adaptive switching strategy is more complicated. Additionally, in each work mode, the pulse parameters can be changed in real time to improve radar’s performance or survivability based on environment detecting [2,3]. In such context, the effectiveness of conventional jamming decision-making methods is decreasing because of a large shortage of prior knowledge about the radars [4]. Therefore, it is urgently necessary to develop radar jamming technology.

Inspired by the cognitive radio, the application of intelligent algorithms in radar confrontation became possible [1,5,6,7]. To solve the problem of the low rate of template matching under the incomplete jamming rule library condition, a jamming decision-making method based on clustering and resampling–support vector machine is proposed in [1]. In [5], a discrete dynamic Bayesian network is established to guide decision-making in self-defense electronic jamming. In [6], an improved chaos genetic algorithm is applied to the allocation of interference strategy. In [7], a particle swarm optimization algorithm is applied to solving optimal jamming power allocation strategy aiming at cognitive MIMO radar. However, these traditional machine learning methods often require large amounts of tagged radar data acquired in advance, which are difficult to be obtained in actual scenarios.

Up to now, there have been many works on the application of reinforcement learning (RL) in communication jamming and anti-jamming [8,9,10,11,12], providing new ideas for radar confrontation. RL is a type of machine learning technology, where an agent learns from interacting with the environment and takes maximizing the feedback from the environment as its learning goal [13]. In [14], the framework of intelligent jamming based on RL is described, where the cognitive jammer and the radar are respectively regarded as the agent and the environment. In [4], the conversion between different radar working states is modeled, where an RL model is trained to choose the jamming mode against each radar state. A frequency-agile radar is considered in [15], and a jamming frequency selection algorithm based on Q-learning is proposed to solve the optimal frequency for each jamming pulse. In [16,17,18,19], other methods based on RL are used to optimize the strategy of combating jamming for radar. Compared with the traditional machine learning methods mentioned above, the agent in RL can learn with no tagged data needed, which makes it more adaptable to the unknown environment. The jamming system equipped with RL can obtain training samples during the jamming process, and update the jamming strategy dynamically based on the change of the radar signal.

However, in the existing work of jamming decision-making based on RL, two common measures of the adaptive radar including mode switching and parameter agility have not been jointly considered. For example, [14] focuses on macro-level modeling of radar jamming, abstracting radar modes into the environment state in RL. What is not noticed is that the jamming effectiveness will be weakened due to the agile actions such as frequency agility [16] and dynamic pulse repetition interval [20] taken by radar in each mode. In [15], the authors aim at the frequency hopping of the radar without considering multiple work modes. According to the authors of [3], if only static behavior of radar is considered, such as a single work mode, it is easy to result in a subjective or local optimal jamming strategy. Moreover, when facing the adaptive radar with multi-modes and the ability of parameter agility, a large jamming action space is usually needed to ensure that the correct actions are included, which greatly increases the complexity of jamming parameters generating. As high complexity will lead to a long convergence time, the jammer can hardly find the optimal jamming strategy in a limited time, which can be fatal to the protected target.

To overcome the problems above, a two-level jamming decision-making framework is developed in this paper. On this basis, a dual Q-learning (DQL) model is proposed to obtain the optimal jamming strategy. Specifically, the jamming decision-making process is disassembled into two levels, where the jamming mode is decided in the first level with the outer Q-learning, and the pulse parameters for the decided jamming mode are selected in the second level with the inner Q-learning. This structure greatly reduces the dimension of the action space of the jammer. With smaller action space and fewer parameters to be learned, it can effectively avoid falling into the local optimum while shortening the convergence time.

Another issue needed to be considered is how to express the feedback from the environment of RL in the jamming decision-making scene. Different from the previous methods where the feedback is statically assigned through the experience matrix, in this paper, we evaluate the effectiveness of jamming as the feedback of the DQL model. On jamming effectiveness evaluation, currently most of the research is based on data collected from the radar side which is impractical due to the non-cooperative nature of the battlefield. However, there are only a few studies on the evaluation from the jamming side. In [21], the accumulated amplitude extracted from signals is chosen as the characteristic statistic to evaluate the jamming effectiveness. In [22], the authors advise using the change of the radar threat level as the basis for the evaluation. In [23], an evaluation method based on feature space weighting in non-cooperative scenes is proposed, where the weight values of evaluation indicators are solved with offline simulated radar data. Considering the variability of the indicator’s contribution to the evaluation result, in this paper, the indicators’ weight values are calculated with their entropy and are updated constantly based on the real-time radar data. With the dynamic weights, the jamming effectiveness is evaluated through measuring the distance between the indicator vectors before and after jamming. The evaluation result is served as the feedback of the environment, based on which the DQL model updates dynamically.

The main contributions of this paper are summarized as follows:

- An RL model named DQL is constructed to guide the jamming decision-making against adaptive radars, where the jamming mode and jamming parameters are hierarchically selected and jointly optimized. Because of the reduced dimensionality of action space, the globally optimal solution can easily be found with a shorter convergence time.
- A new jamming effectiveness evaluation method based on indicator vector space is proposed to serve the feedback to the DQL model, which effectively overcomes the dependence on subjective experience when the model updates. Additionally, in view of the variable electromagnetic environment, the indicators’ weights are calculated dynamically with the real-time radar data, to make the evaluation result more credible.

The rest of this paper is organized as follows. The system model is introduced and the problem of jamming strategy optimization is formulated in Section 2. The proposed DQL model and jamming effectiveness evaluation method are explained in Section 3. The details of the simulations and the analysis of results are shown in Section 4, followed by the conclusion presented in Section 5.

## 2. System Model and Problem Formulation

### 2.1. System Model

**Figure 1:**
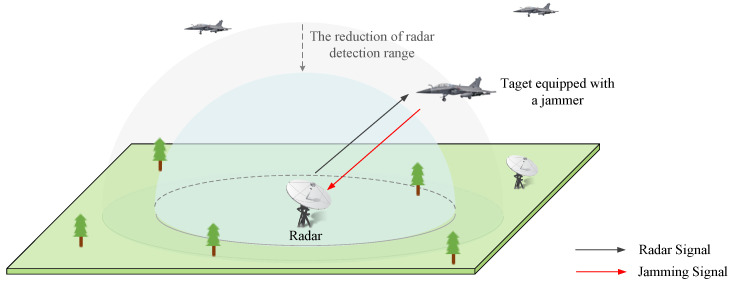
The self-defense electronic jamming scenario: through effective jamming, the jammer can shorten the detection range of the radar to protect the target.

Consider a self-defense electronic jamming scenario, where each target is equipped with a cognitive jammer, shown in Figure 1. Taking a radar and a jammer into account, the jammer devotes to optimizing jamming effectiveness by learning the strategy of the radar, in order to protect the target from detection. The adaptive radar has I work modes such as search, tracking, and guidance, denoted as $\left\{M_{r_{1}},M_{r_{2}},\ldots ,M_{r_{I}}\right\}$. The work modes are switched adaptively between two adjacent beam dwell periods according to the signal-to-jamming radio (SJR). For different modes, the radar changes signal parameters pulse by pulse according to different rules. Similarly, the jammer has J jamming modes such as frequency-spot jamming, blocking jamming, and swept jamming, denoted as $\left\{M_{j_{1}},M_{j_{2}},\ldots ,M_{j_{J}}\right\}$. For different jamming modes, the jammer can select parameters for each jamming pulse.

To simplify the analysis, we regard the target as a point target with radar cross-section (RCS) σ. Assume the radar would be jammed in each beam dwell period, which is called a jamming round in this paper. The number of radar pulses in a jamming round depends on the beam dwell time and the pulse repetition interval (PRI). For the *n*th radar pulse in a jamming round, the carrier frequency (CF) is $f_{r}^{\left(n\right)}$, the bandwidth (BW) is $B_{r}^{\left(n\right)}$, the PRI is $pri_{r}^{\left(n\right)}$ which represents the time between the rising edge of (n−1)th and *n*th radar pulse, the pulse width (PW) is $pw_{r}^{\left(n\right)}$ and the transmission power is $P_{r}^{\left(n\right)}$. At each pulse, the jammer attempts to align the jamming signal with the radar signal in both time and frequency domains. For the *n*th jammer pulse, the CF is $f_{j}^{\left(n\right)}$, the BW is $B_{j}^{\left(n\right)}$, the pulse delay time is $dt_{j}^{\left(n\right)}$ which represents the time from receiving the *n-1*th radar pulse to transmitting next jamming pulse, the PW is $pw_{j}^{\left(n\right)}$ and the transmitting power is $P_{j}^{\left(n\right)}$. In addition, the distance from the radar to the target is *D*, and the wavelength of the radar is λ. The antenna gain of the radar and the jammer is $G_{r}$ and $G_{j}$. The transmission loss of the radar and jamming signal is $L_{r}$ and $L_{j}$. The loss coefficient of polarization matching between the jamming signal and the radar signal is μ. Based on the above definitions, the power of *n*th echo at the radar receiver can be expressed as

(1) $$P_{rs}^{\left(n\right)}=\frac{P_{r}^{\left(n\right)}G_{r}^{2}\sigma \lambda^{2}}{{\left(4\pi \right)}^{3}D^{4}L_{r}}$$

The power of *n*th jamming pulse at the radar receiver is

(2) $$P_{rj}^{\left(n\right)}=\frac{P_{j}^{\left(n\right)}G_{j}G_{r}\lambda^{2}\mu }{{\left(4\pi \right)}^{2}D^{2}L_{j}}$$

Introducing effective jamming coefficient to amend the calculation formula of SJR, the average SJR for *n*th radar pulse $SJR^{\left(n\right)}$ is calculated as:

(3) $$SJR^{\left(n\right)}=\frac{P_{rs}^{\left(n\right)}}{P_{rj}^{\left(n\right)}}\cdot \frac{1}{X_{f}^{\left(n\right)}}\cdot \frac{1}{X_{t}^{\left(n\right)}}=\frac{P_{r}^{\left(n\right)}G_{r}\sigma L_{j}}{P_{j}^{\left(n\right)}G_{j}\mu 4\pi D^{2}L_{r}X_{f}^{\left(n\right)}X_{t}^{\left(n\right)}},$$

where $X_{f}^{\left(n\right)}$ and $X_{t}^{\left(n\right)}$ are the effective jamming coefficients in frequency domain and time domain respectively, expressed as:

(4) $$X_{f}^{\left(n\right)}=\frac{\Delta f^{\left(n\right)}}{B_{j}^{\left(n\right)}}\cdot sgn\left(\Delta f^{\left(n\right)}\right),$$

(5) $$X_{t}^{\left(n\right)}=\frac{\Delta t^{\left(n\right)}}{pw_{j}^{\left(n\right)}}\cdot sgn\left(\Delta t^{\left(n\right)}\right),$$

where $\Delta f^{\left(n\right)}$ and $\Delta t^{\left(n\right)}$ are the overlapping rates in frequency domain and time domain respectively, defined as:

(6) $$\Delta f^{\left(n\right)}=\min\left(f_{j}^{\left(n\right)}+B_{j}^{\left(n\right)}/2,f_{r}^{\left(n\right)}+B_{r}^{\left(n\right)}/2\right)-\max\left(f_{j}^{\left(n\right)}-B_{j}^{\left(n\right)}/2,f_{r}^{\left(n\right)}-B_{r}^{\left(n\right)}/2\right),$$

(7) $$\Delta t^{\left(n\right)}=\min\left(dt_{j}^{\left(n\right)}+pw_{j}^{\left(n\right)},pri_{r}^{\left(n\right)}+pw_{r}^{\left(n\right)}\right)-\max\left(dt_{j}^{\left(n\right)},pri_{r}^{\left(n\right)}\right),$$

where sgn(x)=1 if x>0, otherwise sgn(x)=−1. To reflect the performance of the algorithm more intuitively, we calculate jamming-to-signal radio (JSR) in the simulations to measure the jamming effect, where $JSR^{\left(n\right)}=1/SJR^{\left(n\right)}$.

### 2.2. Problem Formulation

The jamming problem can be modeled as a finite Markov decision process (MDP) [24], expressed as a quaternion {S,A,P,R}. S is a finite set of radar states, where state s∈S is determined by the radar mode and radar pulse parameters. A is a finite set of jammer actions, where action a∈A is defined by the jamming mode and jamming pulse parameters. $P(s^{\left(n+1\right)}|s^{\left(n\right)},a^{\left(n\right)})$ is the transition probability describing how the current state $s^{\left(n\right)}$ transfers to next state $s^{\left(n+1\right)}$ when the jammer takes action $a^{\left(n\right)}$. R is the immediate reward after each action is taken.

Reinforcement learning has been proved to be an effective way to solve MDP problems, the key of which is to find the optimal policy π:S→A to determine which action should be taken at each state. To estimate the effect of a policy, the state-value function for policy π is introduced as:

(8) $$v_{\pi }\left(s\right)=E_{\pi }\left[\sum_{i=0}^{\infty }\gamma^{i}R^{\left(n+i+1\right)}|s^{\left(n\right)}=s\right],$$

where $E_{\pi }\left[\cdot \right]$ stands for expected value with the policy π given. γ∈(0,1] is the discount rate of the reward R, which means that long-term reward is considered and its influence decreases with time. Then, the optimal policy $\pi_{*}$ we aim to find is:

(9) $$\pi_{*}=\arg\max_{\pi }\;v_{\pi }\left(s\right),\forall s\in S$$

## 3. Proposed Jamming Scheme Based on DQL Model

**Figure 2:**
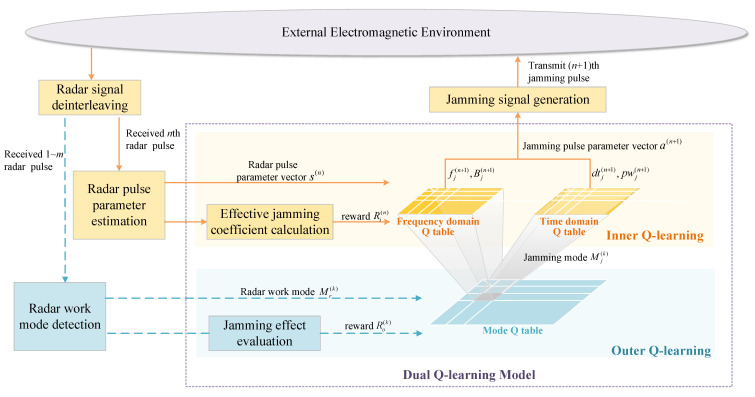
Jamming decision-making based on DQL model: During a jamming round, the radar work mode is discerned with the earliest received *m* radar pulses. Once a new radar mode $M_{r}^{\left(k\right)}$ is recognized, it is sent to the outer Q-learning module. Then the jamming mode $M_{j}^{\left(k\right)}$ is chosen and map to time domain Q table and frequency domain Q table in the inner Q-learning module. According to $M_{r}^{\left(k\right)}$ and $M_{r}^{\left(k-1\right)}$, the jamming effectiveness of last jamming round is evaluated, with which the outer Q table can be updated according to Equation (11). When receiving the *n*th (n>m) radar pulse, the radar pulse parameter vector $s^{\left(n\right)}$ is obtained through parameter estimation. Then the jamming parameters are selected to constitute the parameter vector $a^{\left(n+1\right)}$, and the jamming signal will be generated. According to $s^{\left(n\right)}$, the two effective jamming coefficients are evaluated, with which the inner Q table can be updated according to Equation (13).

To interfere the adaptive radar with mode switching and parameter agility, we propose an RL model named dual Q-learning to optimize jamming strategy, as shown in Figure 2. The jammer’s action space is disassembled into two subspaces containing jamming mode and pulse parameters respectively to reduce the dimensionality, based on which the jamming procedure can be divided into two levels. The jamming mode is determined in the first decision-making level, and specific parameters in frequency and time domain are selected in the second level according to the jamming mode. Two interactive Q-learning models are constructed to find the global optimal solution, and a dynamic method for jamming effectiveness evaluation is designed to obtain the feedback of the DQL model. The interaction between the two levels can be described as: the jamming mode determined in the first level has a guiding effect on the selection in the second level, and the pulse parameters selected in the second level directly determine the SJR at the radar receiver and affect the mode switching of the radar, thereby affecting the next input state of the first level.

### 3.1. Jamming Decision-Making through DQL Model

In the jamming procedure based on the DQL model mentioned above, the outer Q-learning and the inner Q-learning model are trained simultaneously to solve the optimal jamming strategy.

#### 3.1.1. Outer Q-Learning

The outer Q-learning is modeled to acquire the jamming mode in the first decision-making level, where the radar work mode and the jamming mode are regarded as the environment state and the action of the agent, respectively. When obtaining radar work mode $M_{r}^{\left(k\right)}$ at time *k*, the jammer chooses jamming mode $M_{j}^{\left(k\right)}$ as:

(10) $$M_{j}^{\left(k\right)}=\underset{M_{j}}{\arg\max}Q_{o}\left(M_{r}^{\left(k\right)},M_{j}\right)$$

$Q_{o}\left(M_{r}^{\left(k\right)},M_{j}^{\left(k\right)}\right)$ in the outer Q table is updated when the new radar mode $M_{r}^{\left(k+1\right)}$ is obtained at time (k+1) according to the following rule:

(11) $$\begin{array}{cc} \; & Q_{o}\left(M_{r}^{\left(k\right)},M_{j}^{\left(k\right)}\right)\leftarrow Q_{o}\left(M_{r}^{\left(k\right)},M_{j}^{\left(k\right)}\right)\\ \; & +\alpha \left[R_{o}^{\left(k+1\right)}+\gamma \max_{M_{j}}Q_{o}\left(M_{r}^{\left(k+1\right)},M_{j}\right)-Q_{o}\left(M_{r}^{\left(k\right)},M_{j}^{\left(k\right)}\right)\right], \end{array}$$

where α∈(0,1] is the learning rate of the outer Q-learning, which signifies the updating stride of Q value. $R_{o}^{\left(k+1\right)}$ is the reward calculated at time (k+1) for the outer Q-learning, which depends on the evaluation result of jamming effectiveness according to radar mode switching. The evaluation method will be introduced in the next section.

#### 3.1.2. Inner Q-Learning

The inner Q-learning is modeled to solve the optimal jamming parameters in the second decision-making level. The jamming mode selected in the first decision-making level maps to inner Q tables in frequency and time domains. When obtaining the *n*th radar pulse parameter vector s${}^{\left(n\right)}=\left[f_{r}^{\left(n\right)},B_{r}^{\left(n\right)},pri_{r}^{\left(n\right)},pw_{r}^{\left(n\right)},P_{r}^{\left(n\right)}\right]$, the jammer takes $\left[f_{r}^{\left(n\right)},B_{r}^{\left(n\right)}\right]$ and $\left[pri_{r}^{\left(n\right)},pw_{r}^{\left(n\right)}\right]$ as the input states of frequency and time domain Q tables respectively, and the jamming parameters will be chosen according to:

(12) $$p_{j}^{\left(n+1\right)}=\underset{p_{j}}{\arg\max}Q_{i}\left(p_{r}^{\left(n\right)},p_{j}\right),$$

where $p_{r}^{\left(n\right)}=\left[f_{r}^{\left(n\right)},B_{r}^{\left(n\right)}\right]$, $p_{j}^{\left(n\right)}=\left[f_{j}^{\left(n\right)},B_{j}^{\left(n\right)}\right]$ in the frequency domain Q table, $p_{r}^{\left(n\right)}=\left[pri_{r}^{\left(n\right)},pw_{r}^{\left(n\right)}\right]$, $p_{j}^{\left(n\right)}=\left[dt_{j}^{\left(n\right)},pw_{j}^{\left(n\right)}\right]$ in the time domain Q table. The power of jamming signal $P_{j}^{\left(n+1\right)}$ is calculated as $P_{j}^{\left(n+1\right)}=\eta \cdot P_{r}^{\left(n\right)}$, where η varies with different jamming modes. Then the jamming parameter vector a${}^{\left(n+1\right)}=\left[f_{j}^{\left(n+1\right)},B_{j}^{\left(n+1\right)},dt_{j}^{\left(n+1\right)},pw_{j}^{\left(n+1\right)},P_{j}^{\left(n+1\right)}\right]$ can be constituted. After the receiving of *n*th radar pulse, the transmission of next jamming pulse will start after a delay of $dt_{j}^{\left(n+1\right)}$, aiming at the (n+1)th radar pulse. In summary, the jammer predicts the radar parameters one step in advance, in order to interfere the next radar pulse in time. When the (n+1)th radar pulse parameter vector $s^{\left(n+1\right)}$ is obtained, $Q_{i}\left(p_{r}^{\left(n\right)},p_{j}^{\left(n+1\right)}\right)$ in the inner Q table is updated according to the following rule:

(13) $$\begin{array}{cc} \; & Q_{i}\left(p_{r}^{\left(n\right)},p_{j}^{\left(n+1\right)}\right)\leftarrow Q_{i}\left(p_{r}^{\left(n\right)},p_{j}^{\left(n+1\right)}\right)\\ \; & +\alpha \left[R_{i}^{\left(n+1\right)}+\gamma \max_{p_{j}}Q_{i}\left(p_{r}^{\left(n+1\right)},p_{j}\right)-Q_{i}\left(p_{r}^{\left(n\right)},p_{j}^{\left(n+1\right)}\right)\right], \end{array}$$

where $R_{i}^{\left(n+1\right)}$ is the reward of the inner Q-learning calculated with $s^{\left(n+1\right)}$ and $a^{\left(n+1\right)}$. $R_{i}^{\left(n+1\right)}=X_{f}^{\left(n+1\right)}$ for the frequency domain Q table, $R_{i}^{\left(n+1\right)}=X_{t}^{\left(n+1\right)}$ for the time domain Q table.

Both in the outer and inner Q-learning, ε-greedy policy is used to choose the jamming mode or parameters, which is an effective way to balance the exploration and exploitation. In this paper, we define $\varepsilon =e^{-\delta \cdot k}$ which decreases as the count of iterations increases, where *k* represents the number of the iteration and the coefficient δ determines the decay rate. δ is set differently for two decision-making levels: $\delta =\delta_{o}$ for the outer Q-learning and $\delta =\delta_{i}$ for the inner Q-learning. Taking the outer Q-learning as an example, the jammer randomly chooses a jamming mode with probability ε, and chooses the optimal jamming mode $M_{j}^{\left(k\right)}$ according to Equation (10) with probability 1−ε. According to the explanation above, a jamming algorithm based on the DQL model is proposed, and more details are shown in Algorithm 1.

**Figure 3:**
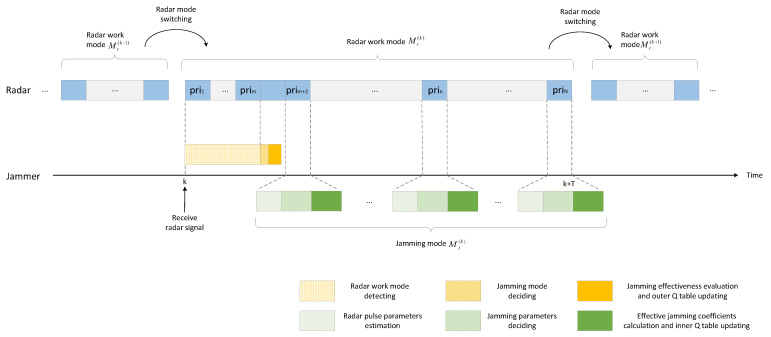
Process of learning and jamming decision making with DQL model.

The time series of learning and jamming decision-making with the DQL model is shown in Figure 3. During a jamming round, the outer Q-learning is performed only once at the beginning to obtain the jamming mode. Under the constraints of the jamming mode, the inner Q-learning is performed in each subsequent PRI to determine the jamming parameters.    
**Algorithm 1:** Jamming algorithm based on DQL model (*K* and $N_{k}$ denote the amount of jamming rounds in simulation and the total number of pulses in the *k*th jamming round respectively. *m* represents the number of pulses required for radar mode discerning.) **for**
k=1,2,…,K
**do**
 
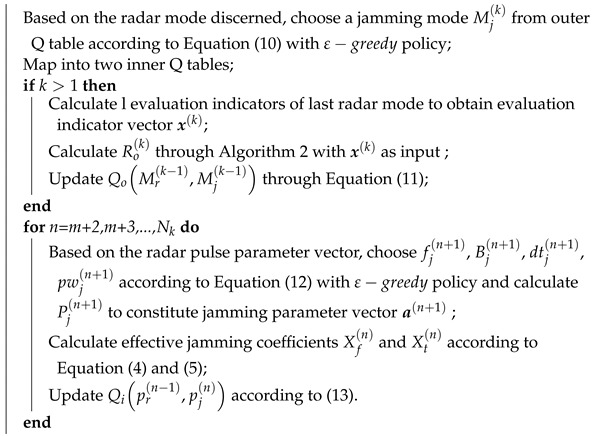
 **end**

### 3.2. Jamming Effectiveness Evaluation through Dynamic Measuring of Vector Distance

Jamming effectiveness evaluation is an important part of the jamming decision-making method based on the DQL model proposed in the previous section. The evaluation result provides the reward for the outer Q-learning. In the radar confrontation, the most intuitive impact of effective jamming on the radar is the reduction of detection probability, which is hard to be known from the jamming side. Therefore, the jamming effectiveness can only be estimated according to the parameters of the radar signal received by the jammer.

To evaluate the jamming effectiveness, we firstly construct an evaluation indicator set $I=\{I_{1},I_{2},\ldots ,I_{l}\}$, where each indicator is a measurable parameter of the radar signal. When the radar is jammed, there are two possible ways for it to change its parameters. One is to switch its work mode. For example, the low SNR causes the radar to lose track of the target, so it shifts from the tracking mode to the search mode. The other possible way is that the radar takes anti-jamming measures in order to improve its performance. For instance, after receiving suppressive jamming, the radar increases the bandwidth to improve its range resolution. Usually, the indicators that can characterize the work mode switching of the radar include beam dwell time, PRI, etc, and the indicators that can characterize the anti-jamming measures taken by the radar include BW, PW, transmitting power, range and speed of frequency agility, etc. The indicators of these two aspects are considered to construct the evaluation indicator set I.

Based on I, a method of vector distance measuring is proposed to calculate jamming effectiveness, where the weight values of evaluation indicators are updated dynamically during the confrontation process. More details are explained as follows.

#### 3.2.1. The Jamming Effectiveness Evaluation Method Based on Vector Distance Measuring

**Figure 4:**
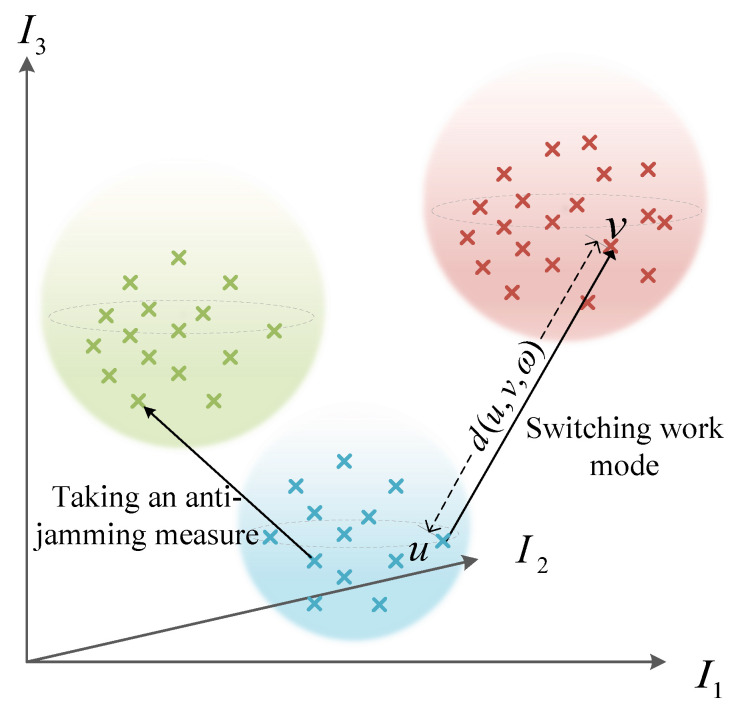
Vector space V${}^{3}$ composed of three evaluation indicators. u, v are two evaluation indicator vectors, and d(u,v,ω) is the Euclidean distance between u and v with weight vector ω.

We construct a *l*-dimensional vector space V${}^{l}$ based on I, where each dimension represents an evaluation indicator. To explain clearly, Figure 4 shows a 3-dimensional vector space of three indicators. As shown, for the same radar state, the evaluation indicator vectors are often clustered together. When the radar switches its working mode or takes anti-jamming measures, the evaluation indicator vector will shift in space. The greater the offset along the increasing direction of the coordinate axes is, the more effective the jamming is. Therefore, the jamming efficiency can be evaluated by measuring the shift of the evaluation indicator vector before and after the jamming.

According to the above analysis, a jamming effect evaluation method based on vector distance measuring is proposed. As known, Euclidean distance can be used to calculate the absolute distance between vectors. We assign a weight for each evaluation indicator on the basis of Euclidean distance, where each weight reflects the contribution of the corresponding indicator to the evaluation result. The calculation formula of Euclidean distance with the indicator weight is expressed as:

(14) $$d\left(u,v,\omega \right)=sgn\left(\sum_{i=1,\ldots ,l}\omega_{i}\left(v_{i}-u_{i}\right)\right){\left(\sum_{i=1,\ldots ,l}\omega_{i}{\left(v_{i}-u_{i}\right)}^{2}\right)}^{\frac{1}{2}},$$

where u, v are two indicator vectors in V${}^{l}$. $\omega =(\omega_{1},\omega_{2},\ldots ,\omega_{l})$ is the weight vector for *l* indicators, $\omega_{i}\in \left(0,1\right)$. $u_{i}$ is the value of *i*th indicator in vector u. $sgn\left(\sum_{i=1,\ldots ,l}\omega_{i}\left(v_{i}-u_{i}\right)\right)$ identifies the symbol of the distance d(u,v,ω), which indicates the direction in which the evaluation indicator vector shifts.

The feedback $R_{o}^{\left(k\right)}$ of the DQL model at time *k* based on jamming effectiveness evaluation is calculated as:

(15) $$R_{o}^{\left(k\right)}=\left\{\begin{array}{c} d\left(x^{\left(k-1\right)},x^{\left(k\right)},\omega^{\left(k\right)}\right)\cdot 10,\;if\;d\left(x^{\left(k-1\right)},x^{\left(k\right)},\omega^{\left(k\right)}\right)\geq 0\;\\ -10,\;\;\;\;\;\;if\;d(x^{\left(k-1\right)},x^{\left(k\right)},\omega^{\left(k\right)})<0 \end{array}\right.,$$

where $x^{\left(k\right)}$ is the normalized evaluation indicator vector obtained at time *k*. $\omega^{\left(k\right)}$ is the weight vector calculated at time *k*, and the calculation method for it is described below.

#### 3.2.2. The Method of Dynamically Weighting for Evaluation Indicators

As the contribution of different indicators to the evaluation result will vary with the change of the radar status, ω is objectively modified through the method of dynamic entropy weight calculation. We horizontally compare the value of each indicator measured in different jamming rounds, and calculate their entropy values to obtain the weights. For each indicator, as a result of the difference between each measurement, its weight is not static, but changes with the received radar signal parameters. Thus, an online evaluation model is established.

For calculating the dynamic entropy weights of evaluation indicators, we define a matrix A $\in R^{l\times m}$, where *m* evaluation indicator vectors can be stored. Once a new radar state is detected, the *l* evaluation indicators of it are calculated and assigned to a column in A. If all the columns in A have been assigned values, the earliest assigned column will be overwritten by the new evaluation indicator vector. $a_{ij}$ in A denotes the value of the *i*th indicator in the *j*th vector. In order to nondimensionalize the calculation and eliminate the impact of different order of magnitudes, the original matrix needs to be normalized to a matrix $B={\left(b_{ij}\right)}_{l\times m}$. The normalization formula is expressed as:

(16) $$b_{ij}=\left\{\begin{array}{c} \frac{a_{ij}-\min\left(a_{ij},\ldots ,a_{in}\right)}{\max\left(a_{ij},\ldots ,a_{in}\right)-\min\left(a_{ij},\ldots ,a_{in}\right)},\;ifI_{i}\propto d\;\\ \frac{\max\left(a_{ij},\ldots ,a_{in}\right)-a_{ij}}{\max\left(a_{ij},\ldots ,a_{in}\right)-\min\left(a_{ij},\ldots ,a_{in}\right)},\;\mathrm{otherwise}. \end{array}\right.,i=1,2,\ldots ,l,j=1,2,\ldots ,m,$$

where $I_{i}\propto d$ indicates that the vector distance *d* is positively correlated to the *i*th indicator in I, which means the lager the value of $\left(v_{i}-u_{i}\right)$ is, the better the jamming effectiveness is. Then the proportion of the *j*th vector for indicator $I_{i}$ is calculated as $p_{ij}=b_{ij}/\sum_{j=1}^{m}b_{ij}$, and the entropy of indicator $I_{i}$ can be calculated as:

(17) $$e_{i}=-\frac{1}{\ln(l)}\sum_{j=1}^{m}p_{ij}\ln(p_{ij}),i=1,2,\ldots ,l$$

Finally, the weight for each indicator is expressed as:

(18) $$\omega_{i}=\frac{1-e_{i}}{\sum_{i=1}^{m}\left(1-e_{i}\right)},i=1,2,\ldots ,l$$

As shown in Figure 2, when a new radar mode is detected at time *k*, the jamming effectiveness of the last jamming round is evaluated, with which $R_{o}^{k}$ is calculated and served as the feedback of the DQL model. The jamming effectiveness evaluation algorithm based on vector distance measuring with dynamic weight is shown in Algorithm 2.
**Algorithm 2:** Jamming effectiveness evaluation algorithm **Input**: Evaluation indicator vector $x^{\left(k\right)}$ **Output**: Jamming effect evaluation result $R_{o}^{\left(k\right)}$ 
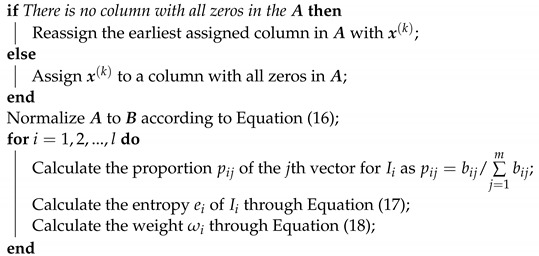
 Calculate the Euclidean distance $d(x^{\left(k-1\right)},x^{\left(k\right)},\omega^{\left(k\right)})$ between normalized $x^{\left(k\right)}$ and  the last indicator vector $x^{\left(k-1\right)}$ with the weight vector $\omega^{\left(k\right)}$ according to Equation (14) Calculate the feedback $R_{o}^{\left(k\right)}$ of the DQL model through Equation (15).

## 4. Numerical Results

To verify the proposed algorithms, a radar parameter template is firstly created, shown in Table 1. Referring to [25,26], four kinds of radar work modes are considered in this paper, including search, acquisition, tracking, and guidance. For the target, the work mode of guidance has the highest threat level, followed by tracking, acquisition, and search. The beam dwell times when the radar is at these four work modes are 80 ms, 100 ms, 120 ms, and 140 ms respectively. For each work mode, two sub-modes are specified with different parameter agility patterns.

The mode switching rule of the radar can be described as: when SJR > −4 db, the radar will raises its threat level; when −7 db < SJR < −4 db, the radar will take anti-jamming measures while maintaining the current work mode, including $M_{r_{1}}\to M_{r_{2}}$, $M_{r_{3}}\to M_{r_{4}}$, $M_{r_{5}}\to M_{r_{6}}$ and $M_{r_{7}}\to M_{r_{8}}$; when SJR < −7 db, the radar will reduce its threat level.

For the jammer, the jamming mode can be switched between $M_{j_{1}}$ and $M_{j_{2}}$, which denote frequency-spot jamming and blocking jamming respectively. The optional jamming parameters for each jamming mode are illustrated in Table 2.

Other parameters in our simulation are given as: $G_{r}=$ 30 dB, $G_{j}$ = 5 dB, $L_{r}$ = 10 dB, $L_{j}$ = 5 dB, *R* = 10 km, $\sigma =1\;m^{2}$. Considering that radar antennas are generally linearly polarized, while jammer antennas are circularly polarized or obliquely polarized, μ is given as 0.5. The parameters in the proposed algorithms are set as: α=0.01, γ=0.8, $\delta_{o}=0.08$, and $\delta_{i}=0.3$. The evaluation indicator set I is constructed of indicators $I_{1}∼I_{7}$, which are shown in Table 3. For each radar mode, the average PRI of all pulses in a period is calculated as indicator PRI, the difference between the maximum and minimum frequency is taken as the range of frequency agility, and the reciprocal of the number of continuous pulses with the same frequency is regarded as the speed of frequency agility.

**Figure 5:**
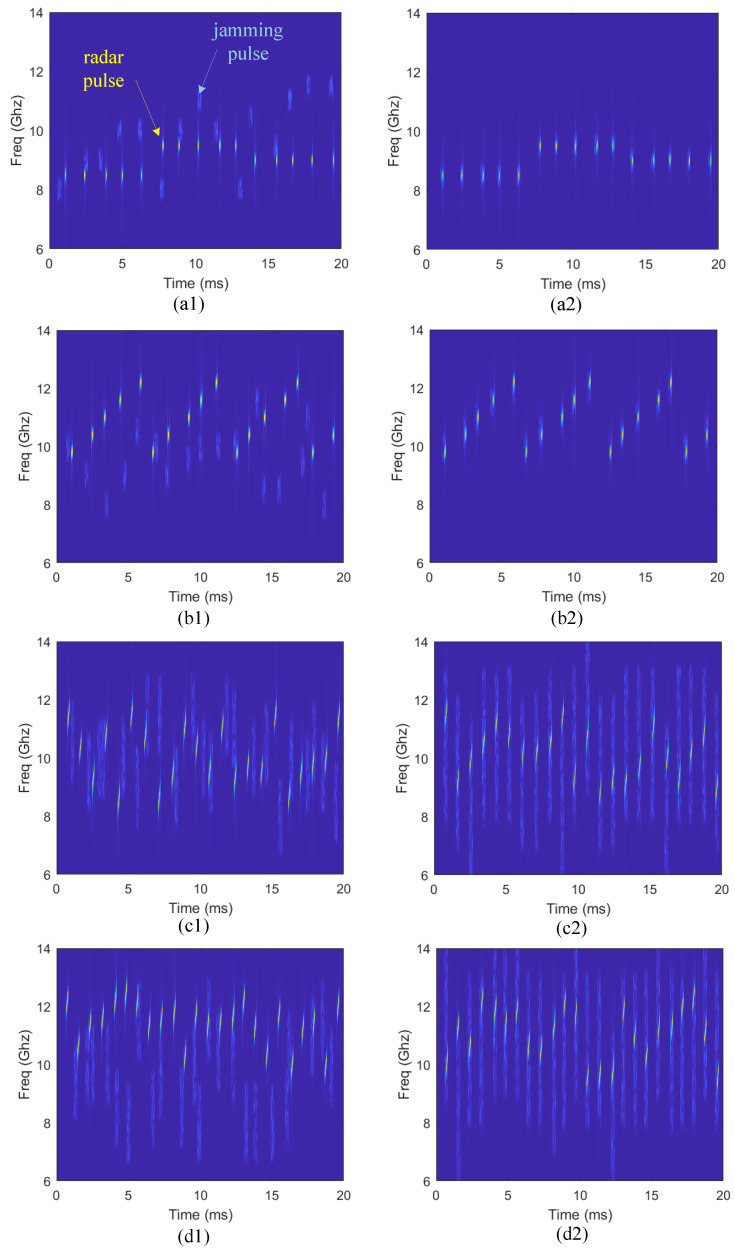
Time-frequency information at the initial and convergent stage: for signals in each image, the brighter strips are radar pulses, the darker strips are jamming pulses. (**a1**,**b1**,**c1**,**d1**) show time-frequency information of radar and jamming pulses at initial stage when the radar is at mode $M_{r_{1}}$, $M_{r_{4}}$, $M_{r_{5}}$, and $M_{r_{7}}$, respectively. (**a2**,**b2**,**c2**,**d2**) show the corresponding time-frequency information at convergent stage for the same four radar modes.

Based on the above description, 200 jamming rounds are simulated. Figure 5 intuitively shows the time-frequency information under four different radar work modes at the initial and convergent stage of learning. Figure 5a1, b1, c1,d1 show the radar and jamming signals at the initial stage, and four other figures show the convergent stage under the same radar work mode. Compared with the random selection of jamming parameters at the initial stage, the jamming pulses can accurately cover radar pulses in time and frequency domains to achieve effective jamming at the convergent stage. Besides, it can be found that if the radar is at modes where the CF changes regularly, the jammer chooses frequency-spot jamming mode $M_{j_{1}}$, otherwise, it chooses to block jamming mode $M_{j_{2}}$, which accords with the common perception.

We compared the performance of our algorithm with the improved chaos genetic algorithm [6], the standard Q-learning [15] and the random parameter selection method. The average JSR of each jamming round is calculated to reflect the jamming effect intuitively. The learning rate and discount rate are set with the same value both in the standard Q-learning and our algorithm. The following results are obtained through 500 independent simulations and their average values are taken to make the figures.

**Figure 6:**
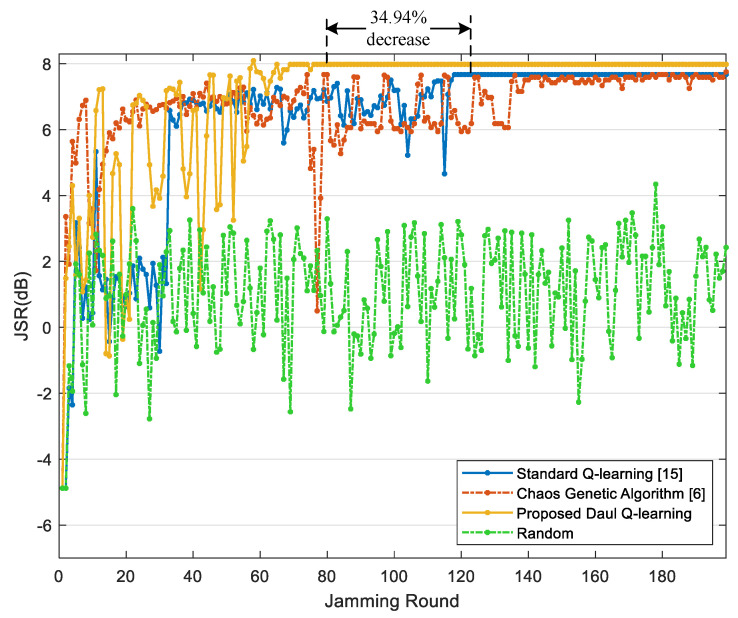
JSR comparison among different methods.

**Figure 7:**
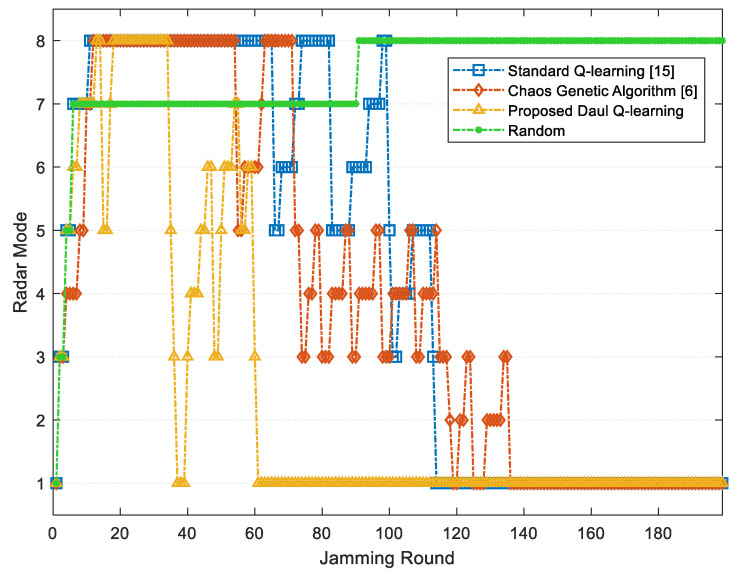
Radar mode switching comparison among different methods: radar modes 1 to 8 represent $M_{r_{1}}$ to $M_{r_{8}}$ respectively.

Figure 6 and Figure 7 respectively show the average JSR and the radar threat level obtained through three methods during 200 jamming rounds. As shown, the improved chaos genetic algorithm [6], the standard Q-learning [15] and the proposed jamming algorithm based on the DQL model can all minimize the threat level of radar, and both the latter two methods can converge and stabilize the JSR within 200 jamming rounds. However, compared with the standard Q-learning, the proposed algorithm can reach the optimal average JSR 7.98 dB, which is 4.05% increased. Further, the convergence time of the proposed jamming algorithm declines by 34.94%, and the number of jamming rounds when the radar is at guidance modes including $M_{r}^{7}$ and $M_{r}^{8}$ reduces by 64.94%. As the high radar threat level is dangerous for the target, the proposed jamming algorithm based on the DQL model can improve the survivability of the target.

**Figure 8:**
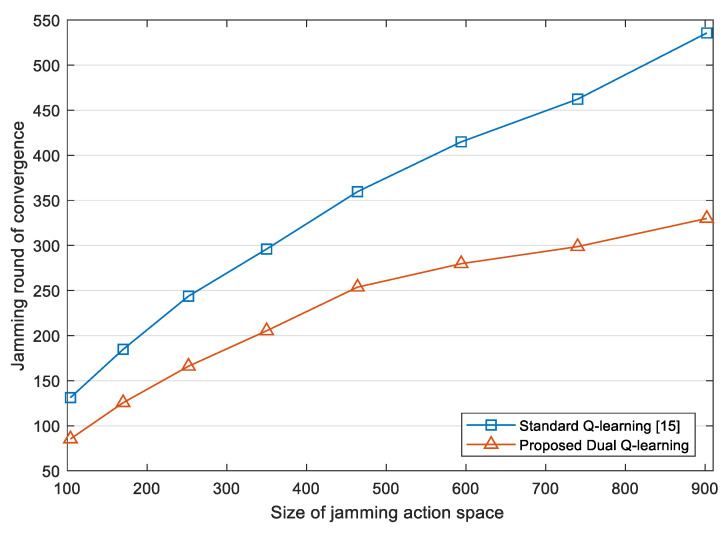
Convergence time comparison among different methods. The size of jamming action space is defined as the product of the total number of optional parameters in time and frequency domains.

In order to further explore the convergence performance of the proposed jamming algorithm, we use the jamming round in which JSR is stable to indicate the convergence time. For jamming round *i*, we calculate the variance of JSR from jamming round (i−19) to jamming round i(i≥20). If the variance is less than 0.01, it is considered that JSR reaches a stable state in jamming round *i*. Figure 8 compares the convergence time of the jamming algorithm based on the standard Q-learning [15] and the proposed DQL model. It is shown that the convergence time of the proposed jamming algorithm is generally lower than that of the standard Q-learning, and as the size of jamming action space increases, the gap between the two grows. Thus, our jamming algorithm based on the DQL model has better scalability, and is more adaptable when larger jamming action space is needed in face of adaptive even unknown radars.

## 5. Conclusions

In this paper, a two-level framework is developed for jamming decision-making against the adaptive radar, and a dual Q-learning model is proposed to optimize the jamming strategy. The jamming mode and pulse parameters are determined hierarchically, greatly reducing the dimensionality of the search space and improving the learning efficiency of the model. In addition, we proposed a new method to calculate the jamming effectiveness by measuring the distance of indicator vectors, where the indicators are dynamically weighted to adapt to the changing environment. The jamming effectiveness evaluation result is served as the feedback value to update the DQL model.

Simulation results show that with the proposed jamming method, the radar joint strategy of mode switching and pulse parameters can be learned within limited interactions, and the optimal jamming effectiveness is reached while the radar’s threat level is minimized. Furthermore, compared with the standard Q-learning, our method improves the average JSR by 4.05% and reduces the convergence time by 34.94%.

It should be emphasized that due to the complex electromagnetic environment, the estimation of radar work mode and pulse parameters is often inaccurate. When there are errors in the input state, the performance of the proposed DQL model will deteriorate. Therefore, in the near future, how to enhance the robustness of the model to deal with the uncertainty of the input is the focus of our investigation.

## Figures and Tables

**Table 1:** Radar parameter template. When the radar is at a certain mode, it selects the pulse parameters according to the rules in the template. Reside and switch, slippery, staggered, and jittered are four of the common radar parameter agility patterns. As shown, reside and switch A:k B:m C:n means that the parameter value stays at A for k pulses, stays at B for m pulses, and stays at C for n pulses; slippery A:B:C means that the parameter value changes from A to C in steps of B; staggered such as [A B C] means that the parameter value is cycled in the order of the list; jittered such as (A, B) means the parameter value is randomly selected from the range of A to B.

Work Mode	Sub-Mode	$f_{r}$/MHz	$B_{r}$/MHz	${\mathrm{pri}}_{r}$/us	${\mathrm{pw}}_{r}$/us	$P_{r}$/kW
search	$M_{r_{1}}$	reside and switch: 8500:5 9500:5 9000:5	100	staggered: [1100 1320 1470]	80	120
	$M_{r_{2}}$	reside and switch: 8600:3 9600:3 9100:3	100	staggered: [1100 1320 1470]	120	120
acquisition	$M_{r_{3}}$	slippery: 8800:600:10000	150	staggered: [1070 1430 857]	120	170
	$M_{r_{4}}$	slippery: 9800:600:12200	150	staggered: [1070 1430 857]	120	170
tracking	$M_{r_{5}}$	jittered: (8500,11500)	800	reside and switch: 830:2 890:4 960:3	120	170
	$M_{r_{6}}$	jittered: (7500,12500)	1000	reside and switch: 830:2 890:4 960:3	120	170
guidance	$M_{r_{7}}$	jittered: (9500,12500)	800	slippery: 740:40:900	120	200
	$M_{r_{8}}$	jittered: (8500,13500)	1000	slippery: 740:40:900	120	200

**Table 2:** Jamming parameter template. When a jamming mode is determined, the corresponding pulse parameters are selected according to the rules in the template. {A:B:C} denotes a set of optional values, consisting of an arithmetic sequence from A to C with B as the difference.

Mode	$f_{j}$/MHz	$B_{j}$($\times B_{r}$)	${\mathrm{dt}}_{j}$/us	${\mathrm{pw}}_{j}$($\times {\mathrm{pw}}_{r}$)	$P_{j}$($\times P_{r}$)
$M_{j_{1}}$	{8000:500:12000}	3	{800:100:1500}	2	0.003
$M_{j_{2}}$	{8500:1000:11500}	6	{800:100:1500}	2	0.006

**Table 3:** Jamming effectiveness evaluation indicator set. The correlation to evaluation result is positive means that the greater the increase of this indicator, the better the jamming effectiveness. While the negative attribute means that the greater the decrease of this indicator, the better the jamming effectiveness.

Evaluation Indicator	Explanation	Correlation to Evaluation Result
$I_{1}$	PRI	positive
$I_{2}$	power	positive
$I_{3}$	beam dwell time	negative
$I_{4}$	BW	positive
$I_{5}$	PW	positive
$I_{6}$	range of frequency agility	positive
$I_{7}$	speed of frequency agility	positive
